# Tuning the Electrical and Thermoelectric Properties of N Ion Implanted SrTiO_3_ Thin Films and Their Conduction Mechanisms

**DOI:** 10.1038/s41598-019-51079-y

**Published:** 2019-10-09

**Authors:** Anuradha Bhogra, Anha Masarrat, Ramcharan Meena, Dilruba Hasina, Manju Bala, Chung-Li Dong, Chi-Liang Chen, Tapobrata Som, Ashish Kumar, Asokan Kandasami

**Affiliations:** 1 Inter-University Accelerator Centre, Aruna Asaf Ali Marg, New Delhi, 110067 India; 2 Department of Physics, Jamia Milia Islamia University, New Delhi, 110025 India; 3 Institute of Physics, Bhubaneswar, 751005 India; 4 Department of Physics and Astrophysics, Delhi University, New Delhi, 110016 India; 5 Research Center for X-ray Science, Department of Physics, Tamkang University, Tamsui, 251 Taiwan; 6 National Synchrotron Radiation Research Centre, Hsinchu, Taiwan

**Keywords:** Thermoelectric devices and materials, Geothermal energy, Devices for energy harvesting, Thermoelectrics, Electronic properties and materials, Semiconductors, Surfaces, interfaces and thin films, Electronic devices

## Abstract

The SrTiO_3_ thin films were fabricated by pulsed laser deposition. Subsequently ion implantation with 60 keV N ions at two different fluences 1 × 10^16^ and 5 × 10^16^ ions/cm^2^ and followed by annealing was carried out. Thin films were then characterized for electronic structure, morphology and transport properties. X-ray absorption spectroscopy reveals the local distortion of TiO_6_ octahedra and introduction of oxygen vacancies due to N implantation. The electrical and thermoelectric properties of these films were measured as a function of temperature to understand the conduction and scattering mechanisms. It is observed that the electrical conductivity and Seebeck coefficient (*S*) of these films are significantly enhanced for higher N ion fluence. The temperature dependent electrical resistivity has been analysed in the temperature range of 80–400 K, using various conduction mechanisms and fitted with band conduction, near neighbour hopping (NNH) and variable range hopping (VRH) models. It is revealed that the band conduction mechanism dominates at high temperature regime and in low temperature regime, there is a crossover between NNH and VRH. The *S* has been analysed using the relaxation time approximation model and dispersive transport mechanism in the temperature range of 300–400 K. Due to improvement in electrical conductivity and thermopower, the power factor is enhanced to 15 µWm^−1^ K^−2^ at 400 K at the higher ion fluence which is in the order of ten times higher as compared to the pristine films. This study suggests that ion beam can be used as an effective technique to selectively alter the electrical transport properties of oxide thermoelectric materials.

## Introduction

Increasing demand for the clean energy resources led to an alternative approach of environment friendly high performance thermoelectric devices to reuse the waste heat via solid state refrigeration and power generation^1^. Thermoelectric (TE) devices can be used to convert a temperature difference into electricity and vice-versa. Despite their several advantages such as small size, portability and scalability etc^1^., their low efficiency makes it difficult to meet the basic requirement for a practical application like power generation and cooling. The thermoelectric performance is related to a dimensionless figure of merit ZT = *S*^2^σT/κ where, T, *S*, σ and κ represent absolute temperature, Seebeck coefficient, electrical conductivity and thermal conductivity, respectively. The optimization of ZT is challenging due to inter-related material properties like σ, κ. Recent studies have been focussing on SnSe, Bi_2_Te_3_, skutterudites, half heusler, clathrates as thermoelectric materials and have been explored for commercial purposes^2,3^. However, these materials are toxic, expensive, and rare in nature which is an important concern for practical use and large scale commercialization. Due to these limitations, oxide TE materials have received renowned attention for mid- and high temperature thermoelectric applications based on their thermal and chemical stabilities in air and at high temperatures^4^. Among oxide TE, the layered cobalt oxides, such as Na_x_CoO_2_ and Ca_3_Co_4_O_9_ (CO-349), are known to be good *p*-type TE materials^5^ while *n*-type oxides are relatively unavailable. The most promising candidates for *n*-type oxide TE materials include perovskite-type SrTiO_3_ (STO) and CaMnO_3_ ^6^.

Different physical approaches such as band engineering, nanostructuring, low dimensionality, superlattices and mechanism based strategies like resonant levels, band convergence, and spin Seebeck effect^7,8^ so on have been adopted to improve the ZT of these materials. Ion beam technique is one of the conventional tools in semiconductors to introduce dopants and defects in a controlled way by selecting strategically suitable energies and ion doses. This technique provides independent control of dose and penetration depth. However, ion implantation has been scarcely explored in thermoelectric materials^9,10^. It is reported that ion beam technique can contribute in the modification of lattice thermal conductivity effectively by radiation induced defects engineering or nanostructuring^11,12^. Thus, this technique allows a new approach for improvement of thermoelectric efficiency and requires more studies.

In the present work, we focus on perovskite material STO which is increasingly recognised as *n*-type thermoelectric material because of its unique electronic structure and tunability^13–15^. It has a wide band gap of 3.2 eV and exhibits good thermoelectric properties due to heavy 3*d* electrons^16,17^. Ohta and co-authors have reported that low dimensionality in STO can greatly enhance the *S* as large as five times compared to the bulk system^18^. An extremely high ZT of ∼2.4 at 300 K has been achieved for Nb doped STO thin layer attributed to a two dimensional electron gas layer (2DEG) formed resulting in quantum confinement effect^19,20^. In 2007, Liu *et al*. reported a reduction in resistivity of STO films due to N ion implantation^21^. Literature survey shows that extensive studies have been carried out on the electric and thermoelectric properties of STO^13^. However, there is no previous study on the effect of ion implantation induced defects on the electrical and thermoelectric properties. This study focuses on the effect of low energy N ion implantation on the crystal and electronic structures, electrical and thermoelectric properties of STO. In this work, we also attempt to determine the possible conduction mechanisms at various temperature ranges.

## Results and Discussion

All the films were deposited by pulsed laser deposition on Si substrate using STO target and implanted with 60 keV N ions with fluence; 1 × 10^16^ and 5 × 10^16^ ions/cm^2^. Post implantation vacuum annealing was performed at 700 °C for 2 h. For simplicity, hereafter, annealed films of pristine, 1 × 10^16^ ions/cm^2^ and 5 × 10^16^ ions/cm^2^ are referred as STO, STO-N116, and STO-N516, respectively.

**Figure 1:**
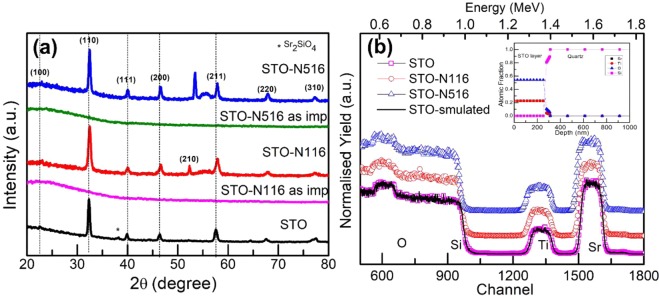
(**a**) X-ray diffraction patterns of STO thin films: STO, STO-N116 and STO-N516. The as-implanted samples for ion fluences 1 × 10^16^ and 5 × 10^16^ ion/cm^2^ show amorphous nature. **(b)** RBS spectra of STO films: STO, STO-N116 and STO-N516. The inset shows the depth profile of STO film as determined from X-RUMP simulation.

Figure 1(a) shows the XRD spectra of pristine and N implanted STO films. Implantation of 60 keV N+ ions resulted in amorphization in the top layers of STO film as probed by XRD and hence these films were subjected to vacuum annealing of 2 hrs. After annealing, a crystalline phase evolves and gets more pronounced at higher fluence. All the STO films show an intense, sharp reflections assigned to planes of (110), (111), (200), (220) and (310) matching with the JCPDS No. 03–0769 and confirm the formation of cubic perovskite phase. However, a very small silicate phase is observed in pristine sample only. As evident from the XRD, this phase is absent after ion implantation. Hence, silicate is not expected to play a major role in the electrical properties and Seebeck coefficient of N ion implanted STO. The Rietveld refinement has been carried out to calculate the lattice parameters. The calculated lattice parameter of STO is comparable to the near stoichiometric samples about 3.905 nm^22^. After N implantation, the lattice parameter changes slightly and remains almost the same for both the STO-N116 and STO-N516. No significant change is observed since the concentration is very low. The bond length of pristine sample calculated from the Rietveld analysis is found to be in accordance with the literature^23^. It is observed that the bond lengths Ti-O in TiO_6_ octahedra are almost constant after implantation (see Supplementary Information). A small shift in all the diffracted peaks is observed due to the presence of lattice strain arising from the ion implantation. Figure 1(b) shows the experimental RBS spectra of STO-P, STO-N116 and STO-N516 along with the simulated spectrum of STO-P using XRUMP Software^24^. The average thickness is estimated to be 280 nm which is close to the measured value. The inset in Fig. 1(b) shows the atomic fraction of elements as a function of thickness and the ratio of elements Sr:Ti:O is found to be 1:1:2.4. However, this value is only indicative and not absolute as RBS is less sensitive to O element.

**Figure 2:**
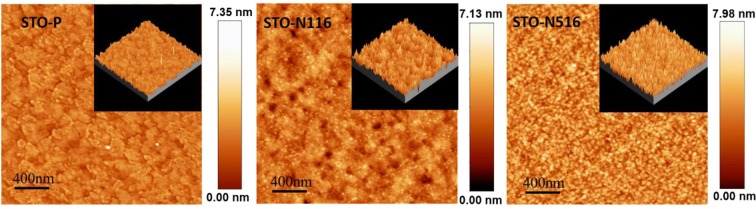
2D-AFM images of STO films: STO, STO-N116, and STO-N516 (size-2 × 2 µm^2^). The inset shows 3D-AFM image of respective films.

Figure 2 shows the AFM images of the STO pristine and N ion implanted thin films. The surface root mean square (RMS) roughness of the pristine film is small (0.94 nm) and after N ion implantation, there is an increase in surface roughness and decrease in grain size from 124 to 114 and 81 nm for STO-N116 and STO-N516 respectively.

**Figure 3:**
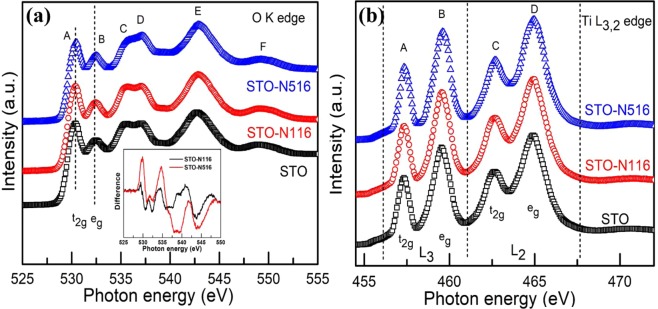
The X-ray absorption spectra of STO, STO-N116 and STO-N516 samples at **(a)** O K-edge and **(b)** Ti L-edge of STO, STO-N116 and STO-N516 samples. The Inset in Fig. 3(a) shows the difference spectra of O K-edge obtained by subtracting the implanted films from the STO spectrum.

Figure 3 show the X-ray absorption spectra at O K- and Ti L-edges corresponding the transitions from core level to unoccupied states following dipole selection rules. The O K edge spectra of pristine and implanted films are shown in Fig. 3(a). The O K-edge features of STO are labelled as A, B, C, D, E and F. The first two main features, A and B, refer as t_2g_ and e_g_ states corresponding to the transitions from O 1 s orbital to O 2p_Π_ and O 2p_σ_ states hybridized with Ti 3d orbitals respectively. The features, C and D are assigned to the transitions from O 1 s orbital to the unoccupied O 2p orbital hybridized with the Sr 4d, and E and F to the Sr 5sp and Ti 4sp orbitals respectively. The peak intensity in O K-edge is related to the number of unoccupied 2p orbitals and also reflects the hybridization with Ti 3d and Ti 4sp orbitals^25^. The crystal field splittings measured from the difference of t_2g_ and e_g_ is 2.06 eV for pristine which is smaller than the bulk value^26^ and it decreases with the N implantation. This is attributed to the small Ti 3d-O 2p overlap resulting in small degree of hybridization^26^. In addition, the intensity ratio of t_2g_ to e_g_ changes with the ion implantation. For high N fluence, The t_2g_/e_g_ ratio decreases as compared to the STO and STO-N516. Apart from this, there is a change in the relative intensities of C and D for STO-N516. Similar behaviour is observed by Mi *et al*.^27^. This change might be related to the distortion that occurs in the TiO_6_ octahedra in STO lattice^28^. Inset in Fig. 3(a) shows the difference spectra obtained by subtracting from the STO. From the difference spectra, it is evident that the STO has significant density of unoccupied states compared to STO-N116 and STO-N516. In other words, the density of unoccupied states in implanted samples decreases. This implies that by N ion implantation, the density of states increases.

The Ti L edge spectra of N implanted STO samples are shown in Fig. 3(b). The spectra at Ti L_3,2_ edges correspond to the transition from 2p^6^ 3d^n^ to 2p^5^3d^n+1^. Here, in case of STO n is zero. Thus, Ti L_3,2_-edges provide the direct information of the unoccupied 3d orbitals and sensitive to the local symmetry^25,27^. In this figure, the four spectral features, A, B, C and D, are assigned to the dipole transitions from Ti 2p_3/2_ (L_3_) and 2p_1/2_ (L_2_) orbitals split due to spin-orbit interaction to unoccupied 3d-orbitals, respectively. Out of these, features A & C correspond to t_2g_ and B & D to e_g_ orbitals^27^. In general, the spectral features of Ti L_2_-edge are very broad compared to Ti L_3_-edge. This is due to the much shorter lifetime of 2p_1/2_ core holes via Coster−Kronig decay^29^. It is observed that there is noticeable broadening in the overall spectra of Ti L-edges which is attributed either to the nitrogen occupancy or defects introduced during N ion implantation. Further, the relative intensities of t_2g_ and e_g_ sub-bands are estimated from the fitting and it is observed that the ratio t_2g_/e_g_ of L_3_ orbital of STO-N116 and STO-N516 decreases as compared to the pristine sample. This indicates that there is local distortion and symmetry of Ti ions indicating change in the hybridization of Ti 3d and O 2p orbitals consistent with the O K-edge.

**Figure 4:**
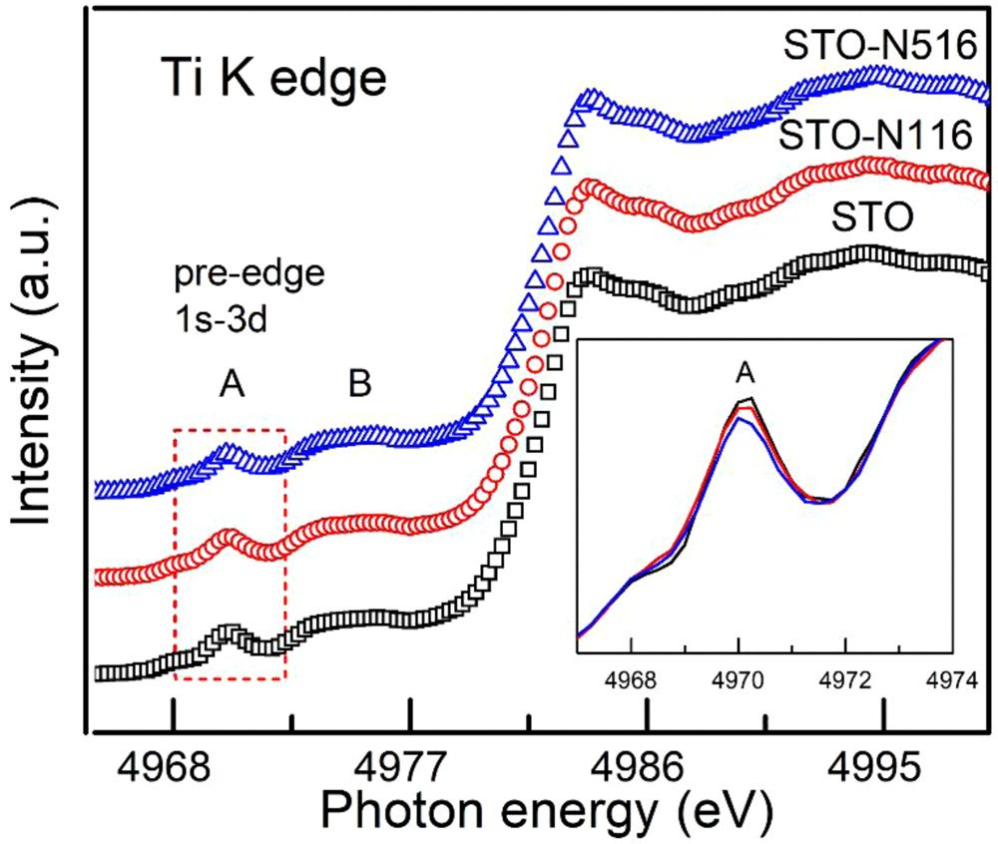
The spectra at Ti K edge of STO, STO-N116 and STO-N516. Inset shows the magnified view of pre-edge region assigned to 1s-3d transition.

Figure 4 shows the X-ray absorption spectra at Ti K-edge of all the samples. The main peak in the spectrum at Ti K-edge corresponds to dipole allowed 1 s to 4p transitions. The feature marked as A is known as pre-edge corresponds to 1s-3d quadrupole transition. The pre-edge region (see Inset of Fig. 4(b)) is the very sensitive to the displacement of Ti atom from its centro-symmetric position in TiO_6_ octahedron^30^. The overall spectra of Ti K edges do not show any drastic change after N ion implantation. As observed in Fig. 4, the pre-edge peak intensity diminishes with the N implantation^29^. The reduction in pre-edge peak intensity is large for STO-N516 as compared to STO-N116 and the pristine films. These result is consistent with the Ti L- and O K-edge data.

**Figure 5:**
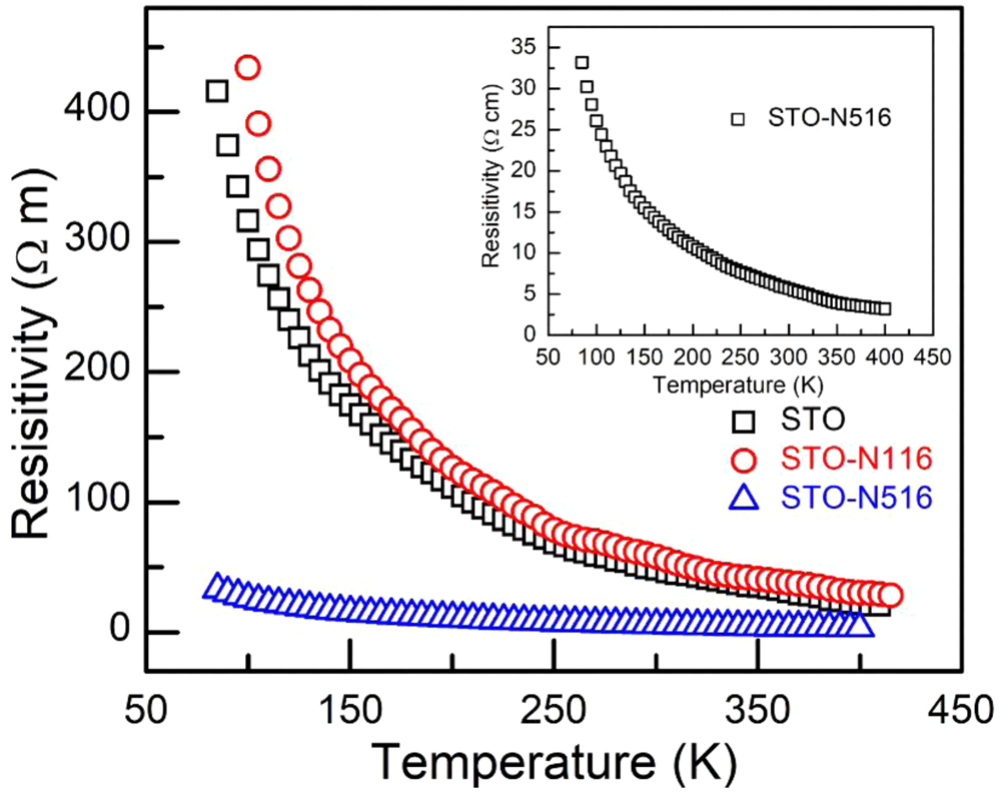
Temperature dependence of resistivity of pristine and N implanted STO thin films. All the films show semiconducting behaviour with temperature. Inset shows the resistivity of STO-N516 with temperature.

To understand the electrical and thermoelectric properties, the temperature dependent resistivity and Seebeck co-efficient were measured. Figure 5 shows the resistivity of pristine and implanted films in the temperature range of 80–400 K. It is evident that the resistivity decreases with temperature indicating a typical semiconducting behaviour in all samples. For the film STO-N116, the resistivity increases as compared to the STO for the entire temperature range. At higher fluence, STO-N516, there is a significant reduction in resistivity compared to the pristine and STO-N116. This reduction is more visible, in particular, at low temperature.

**Figure 6:**
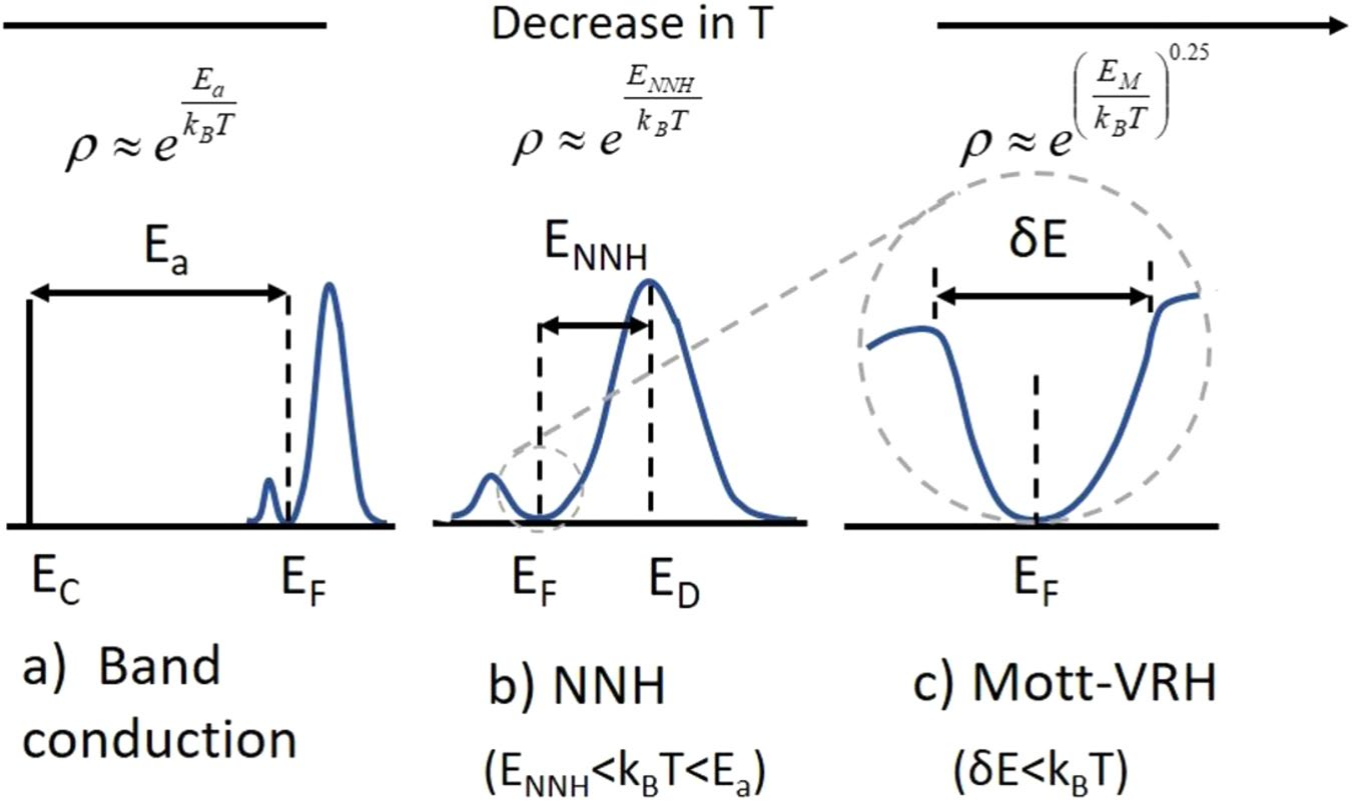
Schematics showing the conduction mechanisms as a function of temperature. **(a)** Band conduction, **(b)** NNH, and **(c)** VRH (Mott-type).

Generally, for semiconductors, the band conduction governs the charge transport properties at high temperature^31^. In band conduction, charge carriers from localized states are thermally activated and transported to the delocalized states. However, in deformed or disordered materials at relatively low temperature, the charge carriers hop through localized states without excitation to the conduction band. Overall, there are mainly two types of conduction mechanisms in such materials, namely (a) band conduction or thermal activation process (b) hopping. This hopping mechanism can be further subdivided into two types: (i) nearest-neighbour-hopping (NNH) and (ii) variable-range-hopping (VRH) as shown in the schematic diagram, Fig. 6. The universal equation governing these conduction mechanisms in semiconductors is given by^31^

1 $$\rho (E)={\rho }_{o}\,\exp [{(\frac{{E}_{t}}{{k}_{B}T})}^{P}]$$

where *ρ*_*ο*_ is the resistivity coefficient, *E*_t_ is the transition energy, *k*_B_ is the Boltzmann constant and *P* (>0) is the characteristic exponent. The exponent *P* defines a different kinds of mechanisms based upon the density of states at Fermi level^31^. For band conduction *P* = 1, and for variable range hopping *P* lies between 0 and 1^32^.

**Figure 7:**
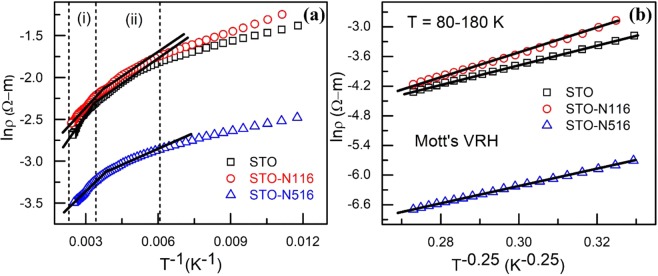
(**a**) plot of lnρ vs. 1/T fitted using (i) band conduction model and (ii) NNH (180–300 K). The crossover temperature from band conduction to NNH is 333 K for STO and 285 K for STO-N116 and STO-N516. **(b)** Plot of lnρ vs. T^−1/4^ fitted using the VRH model. The solid black lines represent the regimes of the corresponding mechanisms.

The experimental data are first analysed using the band conduction model where the exponent *P* = 1 and *E*_t_ corresponds to thermal activation energy (*E*_a_) (Eq. (1)). It is observed that the resistivity data fits well linearly for the pristine film in the temperature range 333–410 K and for STO-N116 and STO-N516 from 285 to 410 K. This suggests that the existence of band conduction mechanism at high temperature regime. The thermal activation energy (*E*_a_) required for the transportation of charge carriers to the delocalized states can be derived by a simple Arrhenius law and it is given in Table 1. Figure 7(a) shows the Arrhenius plot of ln(ρ) vs. 1/T and *E*_a_ is calculated from the slope. The thermal *E*_a_ for pristine STO is derived to be 100 meV which is comparable to the reported experimental results^33^. It is found that the *E*_a_ is found to decrease significantly after N ion implantation, i.e., in STO-N116 and STO-N516 as compared to the STO.

**Table 1:** Transport parameters calculated in the temperature range (80–400 K).

Films	E_s_ (meV) (300–400 K)	E_a_ (meV) (280–410 K)	E_NNH_ (meV) (160–300 K)	E_M_ (meV) (80–218 K)	T_M_ (K) (80–218 K)	R_M_/a (K^−0.25^)	E_a_-E_s_ (meV)	*s*
STO	20	100	40	0.43 T^3/4^	161928	7.72/(T)^1/4^	101	2.01
STO-N116	32	62	32	0.52 T^3/4^	360888	9.19/(T)^1/4^	90	1.67
STO-N516	17	59	26	0.38 T^3/4^	96388	6.61/(T)^1/4^	78.32	2.66

At relatively low temperature, the experimental data deviate from the Arrhenius law. This indicates that the conduction can no longer be described within the band conduction mechanism. The charge carriers do not have sufficient thermal energy to jump over the delocalized states and it is expected to conduct in the nearest localized states via hopping. In such cases, NNH may be the dominating transport mechanism wherein the exponent *P* still remains within 1 (see Eq. 1) and *E*_t_ represents as the nearest neighbour activation energy *E*_NNH_. In NNH conduction mechanism, the charge carrier hop to the nearest neighbour sites but the activation energy required for hopping to empty sites is smaller than the *E*_a_. The temperature dependence of resistivity in case of NNH is given below as^31^:

2 $$\rho (E)={\rho }_{NNH}\,\exp (\frac{{E}_{NNH}}{{k}_{B}T})$$

where *ρ*_NNH_ is a constant and *E*_NNH_ is the nearest neighbour activation energy.

As shown in Fig. 7(a), there exists a close relationship for NNH mechanism within temperature range 180–300 K. The NNH activation energy is calculated from the plot of lnρ vs. 1/T and listed in Table 1. It is observed that *E*_NNH_ is found to be smaller than *E*_a_ for all the samples and hence suggests the presence of NNH conduction.

On further decrease in temperature (T < 180 K), it is found that neither NNH nor band conduction model fits with the experimental data depicting that there is a crossover of conduction mechanism from the NNH to VRH. In lower temperature regime, the possibility of NNH reduces due to a small number of nearest neighbour empty sites which makes the charge carriers to hop between the localised states close to the Fermi level. In that case, the temperature dependence of resistivity can be better expressed by the Mott’s VRH mechanism which is given as^34^

3 $$\rho ={\rho }_{o}\,\exp {(\frac{{T}_{M}}{T})}^{\frac{1}{4}}$$

where *T*_M_ is the Mott’s temperature and *ρ*_ο_ is the resistivity coefficient. For the temperature, 84–218 K, the Mott’s VRH conduction mechanism is used. The linear fit of lnρ against T^−1/4^ as shown in Fig. 7(b) implies that the Mott’s VRH governs the transport in the low temperature regime. The characteristic Mott’s temperature, *T*_M_, obtained from the slope of the fitted curve and represented as^34^

4 $${T}_{M}=\frac{18}{{k}_{B}{a}^{3}N({E}_{F})}$$

where *a* is localization length and *N(E*_F_) is the density of states at Fermi level. It is clear from this equation that T_M_ is inversely proportional to *N(E*_F_). At higher fluence of N ion implantation, STO-N516, the value of *T*_M_ decreases due to the enhancement of the *N(E*_F_) at the Fermi level. Further, the ratio of average hopping (*R*_M_) distance to localization length (*a*) can be calculated from the fitting parameters. According to Mott-VRH model, the average hopping distance (*R*_M_) must be larger than the localization length *a*, which is given as^34^

5 $$\frac{{R}_{M}}{a}=\frac{3}{8}{(\frac{{T}_{M}}{T})}^{\frac{1}{4}} > 2$$

The calculated values are listed in Table 1. From the fitting, the ratio, *R*_M_*/a*, is found to be more than 2 for all samples in the temperature range (80–180 K). This is imperative that the Mott’s VRH conduction mechanism is valid^34^.

In addition, the Mott hopping energy^34^, *E*_M_, can be evaluated and listed in Table 1:

6 $${E}_{M}=\frac{1}{4}{k}_{B}T{(\frac{{T}_{M}}{T})}^{\frac{1}{4}}$$

Above results show that with N ion implantation in STO, different conduction mechanisms are operative in the entire temperature range of 80–400 K. This suggests that at high temperature (T > 300 K) and mid temperature range (180–300 K), the band conduction and NNH conduction mechanisms, respectively prevail and there is a crossover from NNH to Mott’s VRH in low temperature regimes. These results are similar to that of other oxides like ZnO^35^. It may be noted that there exists no data related to STO in literature.

**Figure 8:**
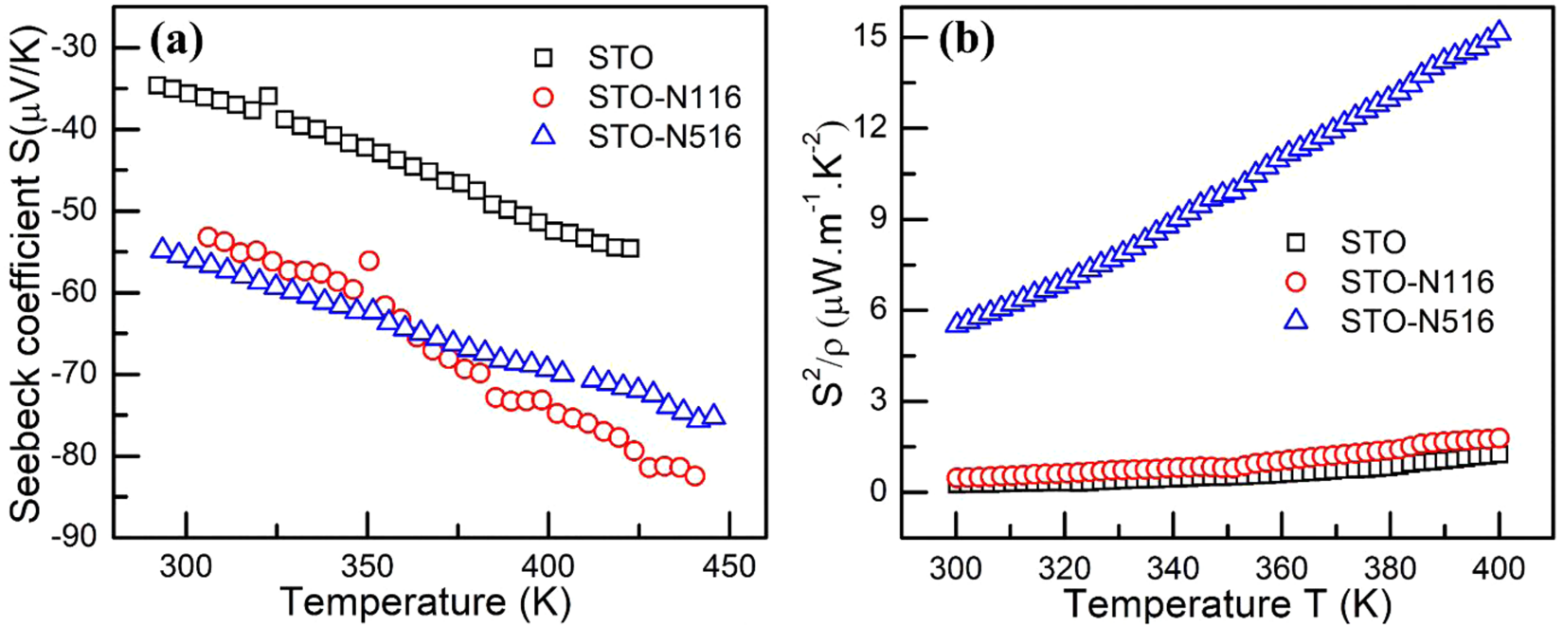
(**a**) Temperature dependence of the *S* of all the films, **(b)** Power factor as a function of temperature in range 300–400 K. The maximum power factor of 15 µWm^−1^ K^−2^ at 400 K for STO-N516.

The Seebeck coefficient is measured in the temperature range of 300–400 K and displayed in Fig. 8(a). The sign of *S* is negative throughout the measured temperature range revealing *n*-type behaviour for all the samples and *S* increases with temperature. The room temperature value of *S* for pristine STO is 35 µV/K which increases to 52 µV/K and 56 µV/K on N implantation with fluence 1 × 10^16^ and 5 × 10^16^ ion/cm^2^, respectively^36^. Fig. 8(b) shows the variation of power factor of N implanted thin films as a function of temperature. The STO-N516 film exhibits the highest power factor of 15 µWm^−1^ K^−2^ at 400 K and this enhancement is related to *S* and the electrical conductivity after N ion implantation.

The temperature dependence of *S* is represented by the Boltzmann’s equation^37^,

7 $$S=-\frac{k}{e}(\frac{{E}_{S}}{{k}_{B}T}+A)$$

where *E*_s_ and *A* are represent as activation energy and scattering parameter for thermopower respectively. The linear dependence of *S* vs. 1000/T is plotted and *E*_s_ is evaluated (see in Table 1). Here, *E*_s_ is represented as the energy difference between the Fermi level and bottom of the conduction band. The derived *E*_s_ is compared with *E*_a_ from the resistivity data in the same temperature range (330–400 K) and found to be smaller than *E*_a_. Using the activation energy determined from the resistivity and thermopower data, one can understand the existence of the localised levels in the band gap and hence successive conduction mechanisms. The concept of large *E*_a_ over *E*_s_ lies in such a way that in the resistivity measurements, the current flows through the film in a closed loop because the charge carriers need to overcome the highest potential barrier during the conduction. However, in thermopower, it is an open circuit measurements wherein the charge carriers have to cross only the local fluctuations^10,38^. The difference ΔE quantifies the magnitude of potential fluctuations. In the present case, the difference is positive suggesting that the thermal activation process governs the conduction in high temperature regime. These observations also support the resistivity data discussed above for the same temperature range of 300–410 K. The difference in the activation energies between both measurements can also be expressed as a dimensionless quantity *Q* given by^39^

8 $$Q=\,\mathrm{ln}\,\sigma -\frac{eS}{k}=C-\frac{\Delta E}{kT}$$

The value of ΔE is estimated from the slope by a linear fit of Q vs 1000/T and found in accordance with the calculated difference *E*_a_ *-E*_s_.

For the detailed understanding of carrier transport, another approach of dispersive transport mechanism can be considered^40^. The dispersive transport is typically associated with conduction through delocalized band states (band conduction). In this model, thermopower –conductivity relation obtained from the Sommerfeld expansion allows one to identify the scattering mechanism by estimating the value of exponent *s* in the following equation^40^.

9 $$S=\frac{{k}_{B}}{q}\frac{{\pi }^{2}}{3}s{(\frac{\sigma }{{\sigma }_{{E}_{O}}})}^{-1/s}$$

where *S*, *σ*, *σ*_*Eο*_, and *s* are Seebeck coefficient, conductivity, transport coefficient in the units of conductivity and transport exponent, respectively. The plot of lnS vs. lnσ is employed to extract *s* from the slope. The value of *s* can be ascribed to different scattering mechanisms, (a) polar-optical phonon scattering (*s* = 2) (b) ionized impurity scattering (*s* = 3) and (c) point defect scattering (*s* = 1)^40^. The calculated value of *s* for pristine STO is 2 which corresponds to polar-optical phonon scattering. For STO-N116 and STO-N516, *s* is 1.67 and 2.66 respectively which suggest that scattering mechanism is changing with the implantation from optical phonon scattering to ionized impurity scattering. The value of *s* for each sample is listed in Table 1.

The ion implantation is a technique that offers precise control of implanted ion/dopant species, profile and temperature. This process involves interaction with materials displacing the host atoms from their original sites and transferring energy to electrons causing ionization and excitations^41^. It can be used to introduce impurities, defects such as oxygen vacancies into lattice, or to investigate the novel properties resulting from the impurity–defect interactions^12,42^. Oxygen deficiency can have a positive effect on the thermoelectric properties. Kumar *et al*. have demonstrated that an increase in concentration of oxygen vacancies enhance simultaneously the carrier concentration and Seebeck coefficient. This results in a large power factor^43^. The main advantages of introducing oxygen vacancies via low-energy ion bombardment over vacuum annealing are (i) the distribution of vacancies can be spatially localized as it is dependent on the penetration depth of the implanted ions and (ii) the absence of secondary phases that occurs during high temperature vacuum annealing leading to distortion of the STO lattice^44^. In the present study, the 60 keV N ion implantation leads to amorphization of few surface layers of STO thin films. Using SRIM software, the nuclear energy loss (S_n_) and electronic energy loss (S_e_) are found to be 11.8 eV/Å and 33 eV/Å respectively. The relative contribution of both these mechanisms induces lattice defects and dislocations causing amorphization as evidenced from the XRD. The post annealing helps in the incorporation of N ions in STO lattice either at interstitial or substitutional sites^27^ and also displaces the atoms to reduce the radiation damage caused by implantation^45^. This leads to stronger crystalline nature. Similar observation has been reported by Kumar *et al*. in case of N ion implanted CeO_2_ ^46^. The X-ray absorption measurements performed to probe the local electronic structure that shows the change in Ti 3d-O2p hybridization and local symmetry resulting from the defects generated by ion implantation. This leads to change in parameters such as crystal field splittings and the t_2g_/e_g_ ratio. This might be attributed to the decrease of transition probability to the empty 3d orbitals due to the reduction in unoccupied orbitals with N occupancy or the defects mainly oxygen vacancy created during implantation. Another reason could be the distortion of TiO_6_ octahedra in STO that modifies the Ti 3d and O 2p hybridization. Hence, this behaviour contributes to the increased conductivity of STO-N516 sample. However, the crystal structure is retained. The AFM images confirm that there is a decrease in the grain size of the films after implantation. Implantation creates defects and vacancies in the lattice, which leads to the increase in the grain boundaries and these in turn scatter the phonons. The films of STO-N516 exhibits large grain boundaries and such increase causes potential barrier scattering resulting in the enhancement of Seebeck coefficient^47^. In addition to this, decrease in crystal size serves as the scattering center of the phonons and hence enhances the Seebeck coefficient and thermal conductivity. This implies that one needs to optimize the crystallite size and oxygen vacancies that control the conductivity and hence achieve maximum ZT.

In the high temperature range, the temperature dependence of resistivity can be explained by band conduction mechanism where the charge carriers have sufficient energy to jump across conduction band. The activation energy calculated from the Arrhenius plot decreases with ion implantation. This may arise due to additional localized states in the band gap. In the mid temperature range, the charge carriers hop to the nearest sites and lowers the activation energy compared to thermal activation energy. As the temperature decreases, the resistivity data deviate from the linear regression of band conduction and NNH mechanisms, and leads to the Mott’s VRH mechanism^34^. At lower temperature, due to insufficient thermal energy, the charge carriers can hop across the localised empty sites at larger distances. The characteristic Mott temperature, *T*_M_ is found to be lowest for high fluence. This results in an enhanced density of states at the Fermi level which is responsible for the increased conductivity. For a better understanding of transport phenomenon, *S* is also analysed by calculating various parameters. The activation energy *E*_*s*_ evaluated from the Boltzmann’s equation is found to be smaller than the thermal activation energy *E*_*a*_ and positive *ΔE* = *E*_*a*_ − *E*_*s*_ suggests the presence of potential fluctuation caused by ion defects. It is found that ion implantation changes the scattering mechanism from optical phonons to defect assisted scattering for STO-N116 sample resulting in a decreased value of *s* (1.67). On further implantation, i.e., STO-N516, doped impurities are sufficiently large in number to contribute and modify the scattering mechanisms which result in ionized impurity scattering with a large value of *s* (2.66). In the present case, the observed scattering centres and localized states in the band gap after implantation might originate from: (a) the defects and distortion in the STO lattice, (b) the oxygen vacancies, and (c) the modification of band structure due to N doping. In other words, the materials having high impurity scattering centres are likely to provide enhanced *S* and power factor. However, a detailed investigation using different ion beams with different oxygen stoichiometry is required to understand the exact common transport and scattering mechanisms and their tunabilities in these materials.

There are a few studies on the first principles calculations using the Density functional theory (DFT) for N doped SrTiO_3_, and TiO_2_ systems assuming the incorporation of N ions such as substitutional, interstitial and oxygen vacancies. The results from Mi *et al*. and Valentin *et al*. reported that the N 2p impurity states are localized and lie slightly above the top of the O 2p valence band^27,48^. They also reported that there is no shift in the position of the O 2p valence band, as well as of the conduction band, with respect to the pristine material. These result in electrons getting trapped easily in the high-energy levels when excited into the conduction band, and responsible for the increased electrical conductivity. On the other hand, Rumaiz *et al*. proposes a bandgap narrowing due to the hybridization of Ti 3d states with unoccupied both O 2p states and N 2p states^49^. The valence-band structure exhibits tailing to lower binding energies due to the incorporation of less tightly bound N 2p level that is hybridized with O 2p level causing a reduction in bandgap.

These first principal calculations can be easily compared with the experimental XAS data. The spectral features of the XAS represent the density of unoccupied states. This is theoretically comparable with band structure calculations that provide projected density of states (DOS)^28^. Inset in Fig. 3(a) shows the difference spectra obtained by subtracting from the STO. From the difference spectra, it is evident that the STO has significant density of unoccupied states compared to STO-N116 and STO-N516. In other words, the density of unoccupied states in implanted samples decreases. This implies that by N ion implantation, the density of states increases. Hence, this increase in DOS is responsible for enhanced power factor.

To conclude, N ion implantation creates defects and vacancies, which affect the transport properties of the carriers. It modifies the transport mechanism by creating trap centers and defects which act as scattering centers^10^. The defects induced leads to the reduction of thermal conductivity and have less effect on S, which in turn improves the ZT value. These observed increase in S could be accounted from the modification of these properties under implantation suggesting scattering of phonons by ionized impurity and vacancies formed in the sample.

## Methods

The STO films (5 × 10 mm^2^) were fabricated by pulsed laser deposition (PLD) system (Excel Instruments, Mumbai) using a KrF excimer laser (248 nm wavelength, 10 Hz repetition rate and 20 ns pulse duration) in Ar atmosphere at a substrate temperature of 700 °C. The p-type Si substrate was used for deposition. Before PLD deposition, only RCA-1 cleaning of substrate was performed and no etching in HF was done to protect the native SiO_2_ layer present on Si substrate^50^. This was confirmed by performing I-V on bare substrates. The STO films were grown by ablation of STO bulk ceramic target at an energy density of 4.2 J/cm^2^ and the target was rotated to ensure uniform deposition. To clean the surface, pre-ablation was done using 500 shots on the target surface. The STO layer was deposited over the insulating SiO_2_ layer. The resistance of the STO films is in kΩ which excludes any contribution from the substrate. This implies that there is no effect from the substrate. The thickness of all films was measured as ~260 nm using Surface Profilometer (Ambios, XP-200, USA). The X-ray diffraction pattern of these STO films exhibit crystalline nature. These films were then implanted with 60 keV N^+^ ion beam with two different fluences, 1 × 10^16^ and 5 × 10^16^ ion/cm^2^ using Low Energy Ion Beam Facility (LEIBF) at Inter University Accelerator Centre (IUAC), New Delhi. The energy of ions is selected to implant N ions within STO layer of thickness 260 nm. The projected range of N ions in STO lattice has been pre-simulated using TRIM (Transport of Ions in Matter) code and is estimated to be ∼102 nm (±40 nm, straggling)^51^. Post implantation vacuum annealing was performed at 700 °C for 2 h. For simplicity, hereafter, annealed films of pristine, 1 × 10^16^ ions/cm^2^ and 5 × 10^16^ ions/cm^2^ referred as STO, STO-N116, and STO-N516, respectively. The equivalent doping concentrations in STO lattice are estimated to be 3.3 × 10^20^ ions/cm^3^ and 1.5 × 10^21^ ions/cm^3^ for STO-N116 and STO-N516 respectively.

The X-ray diffraction (XRD) measurements were performed to determine the structural information at glancing angle (1°) by Philips X’pert PRO (Model PW 3040) diffractometer in the range of 20°–80°. For compositional and thickness measurements, Rutherford backscattering (RBS) spectromentry was performed using 2 MeV H^+^ ions at IUAC, New Delhi. The scattering angle was maintained at 165°. The surface morphology was examined by Multi Mode Scanning Probe Microscopy (Bruker) in tapping mode. In order to investigate the chemical and electronic structural information, Ti L-and O K-edge spectra were recorded in total electron yield (TEY) mode at high-energy spherical grating monochromator (HSGM) beamline 20A1 of the National Synchrotron Radiation Research Centre (NSRRC) in Hsinchu, Taiwan. The energy resolution ΔE/E was of order of 1/5000. The Ti K edge measurements were performed at wiggler beamline 17C in total fluorescence yield (TFY) and Ti foil was used for calibration. The photon energy resolution is 0.3 eV. The *S* and resistivity measurements were performed using an in-house developed experimental set-up^52^.

## Conclusion

In summary, ion implantation studies on PLD deposited STO thin films were carried out to understand the electrical transport and scattering mechanisms. The XRD confirms the cubic perovskite phase of pristine and implanted films. The X-ray absorption spectra show that N implantation affects the local symmetry of TiO_6_ octahedron by generating defects mainly oxygen vacancies. There is an enhancement in electrical conductivity and thermopower at high implantation dose. The electrical transport properties of N ion implanted STO films reveal different conduction mechanisms: the band conduction in the high temperature regime, nearest neighbour and variable range hopping in the low temperature regime attributed to the presence of defect induced localized states in the band gap. The increased power factor (15 µWm^−1^ K^−2^) for higher fluence film depicts the significance of ion implantation technique which provides a new tool for defect engineering in the applications of thermoelectric devices.

## Supplementary information

Supplementary information
