# Supplementary material for: Tuning the Electrical and Thermoelectric Properties of N Ion Implanted SrTiO3 Thin Films and Their Conduction Mechanisms
Source: Sci Rep. 2019 Oct 9;9:14486. doi: 10.1038/s41598-019-51079-y (PMC6785559; doi:10.1038/s41598-019-51079-y)
Supplement: Supplementary file 1 — Supplementary information [file 41598_2019_51079_MOESM1_ESM.pdf]

Supplementary Information: Tuning the Electrical and Thermoelectric Properties by N Ion Implantation in $SrTiO_3$ Thin Films and Their Conduction Mechanisms

Anuradha Bhogra[1*], Anha Masarrat[1,2], Ramcharan Meena[1], Dilruba Hasina[3], Manju Bala[4], Chung-Li Dong[5], Chi-Liang Chen[6], Tapobrata Som[3], Ashish Kumar[1], and Asokan Kandasami[1$]

[1]Inter-University Accelerator Centre, Aruna Asaf Ali Marg, New Delhi-110067, INDIA
[2]Department of Physics, Jamia Milia Islamia University, New Delhi-110025, INDIA
[3]Institute of Physics, Bhubaneswar - 751005, INDIA
[4]Department of Physics and Astrophysics, Delhi University, New Delhi-110016, INDIA
[5] Department of Physics, Tamkang University, Tamsui, Taiwan 251
[6]National Synchrotron Radiation Research Centre, Hsinchu, Taiwan

Sample images

The optical images of STO, STO-N116 and STO-N516 were taken by optical microscope (Axio Scope, Zeiss Inc.) using 10× objective lens. A white-balance filter (Zeiss, Inc.) was also used for visual observation under the natural light condition.

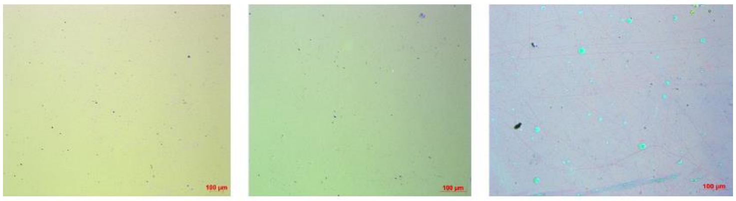

STO-P   STO-N116   STO-N516

TRIM simulations

The energy of ions were selected to implant N ions within STO layer of thickness 260 nm. The projected range of N ions in STO lattice were pre-simulated using TRIM (Transport of Ions in Matter) code (Ziegler et al.) and is estimated to be ~ 102 nm (± 40 nm, straggling).[1] Figure S1 shows the TRIM simulations for N ion distribution in STO film.

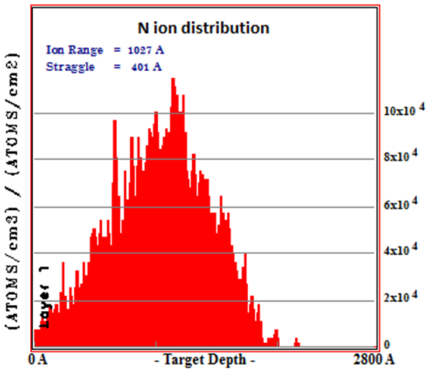

Figure S1. shows the TRIM simulations for N ion distribution in STO film.

Structural properties

The X-ray diffraction (XRD) measurements were performed to determine the structural information at glancing angle (1°) by Philips X'pert PRO (Model PW 3040) diffractometer in the range of 20°-80°. The XRD patterns were refined using Rietveld refinements to calculate the lattice parameters were performed using Match and Fullprof software.[2] However, the N ion concentration is less than 3% even at the implantation of highest fluence of 5E16 ions/cm^2 and most of the N ions are expected to be in the surface as the range of the N ion is 140 nm and also N has high diffusion co-efficient. During vacuum annealing some of the N ions diffuse out from the surface to vacuum as reported by Mi et al.[3] Considering these factors, the % change in lattice parameters is not significant. In addition to lattice parameters, other microscopic parameters like carrier concentration, phonon scattering, crystallite size and defects etc. also determined the thermoelectric properties. The observed and calculated diffraction patterns for all the samples are shown in Fig. S2.

Figure S2 shows the refined and raw data of XRD patterns of STO, STO-N116 and STO-N516. The secondary phase formed in STO is unable to fit using $SrTiO_3$ JCPDS file. This also confirmed the formation of silicates as a secondary phase.

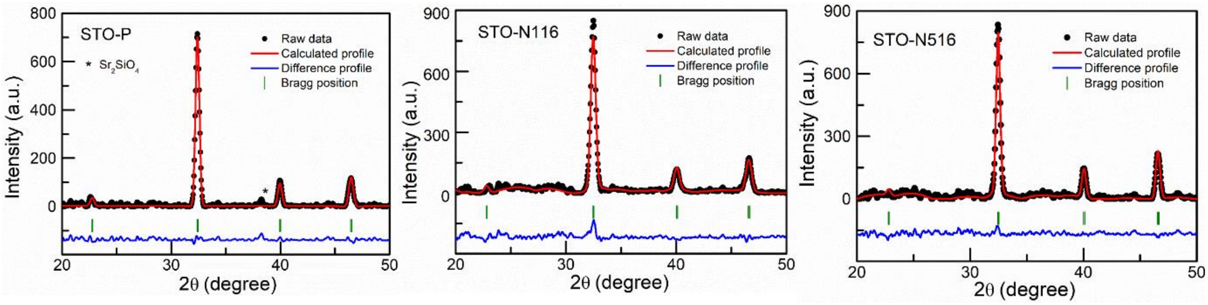

Figure S2. XRD patterns and Rietveld refinement of STO, STO-N116 and STO-N516.

Table S1. Crystal structure parameters and Ti–O bond lengths of STO. STO-N116 and STO-N516 obtained by the Rietveld refinements based on the cubic model.

Films	STO	STO-N116	STO-N516
Lattice parameter (nm)	3.9080(3)	3.899(0)	3.899(2)
Crystallite size (nm)	25.4	16.6	26.1
Ti-O bond length (Å)	1.9540(3)	1.94950(0)	1.9500(11)
GoF (χ^2)	3.43	3.46	4.48

Reference

1 Ziegler, J. F., Ziegler, M. D. & Biersack, J. P. SRIM – The stopping and range of ions in matter (2010). *Nuclear Instruments and Methods in Physics Research Section B: Beam Interactions with Materials and Atoms* **268**, 1818-1823, doi:https://doi.org/10.1016/j.nimb.2010.02.091 (2010).

2 Rietveld, H. A profile refinement method for nuclear and magnetic structures. *Journal of Applied Crystallography* **2**, 65-71, doi:doi:10.1107/S0021889869006558 (1969).

3 Mi, Y. Y. *et al.* Thermal stability of nitrogen-doped SrTiO3 films: Electronic and optical properties studies. *Journal of Applied Physics* **101**, 063708, doi:10.1063/1.2713350 (2007).
